# Structural and Material-Based Approaches for the Fabrication of Stretchable Light-Emitting Diodes

**DOI:** 10.3390/mi15010066

**Published:** 2023-12-28

**Authors:** Hamin Park, Dong Chan Kim

**Affiliations:** 1 Department of Electronic Engineering, Kwangwoon University, 20, Gwangun-ro, Nowon-gu, Seoul 01897, Republic of Korea; 2 Department of Chemical and Biological Engineering, Gachon University, 1342 Seongnam-daero, Sujeong-gu, Seongnam-si 13120, Republic of Korea

**Keywords:** stretchable LEDs, structural approaches, material-based approaches, intrinsically stretchable LEDs

## Abstract

Stretchable displays, capable of freely transforming their shapes, have received significant attention as alternatives to conventional rigid displays, and they are anticipated to provide new opportunities in various human-friendly electronics applications. As a core component of stretchable displays, high-performance stretchable light-emitting diodes (LEDs) have recently emerged. The approaches to fabricate stretchable LEDs are broadly categorized into two groups, namely “structural” and “material-based” approaches, based on the mechanisms to tolerate strain. While structural approaches rely on specially designed geometries to dissipate applied strain, material-based approaches mainly focus on replacing conventional rigid components of LEDs to soft and stretchable materials. Here, we review the latest studies on the fabrication of stretchable LEDs, which is accomplished through these distinctive strategies. First, we introduce representative device designs for efficient strain distribution, encompassing island-bridge structures, wavy buckling, and kirigami-/origami-based structures. For the material-based approaches, we discuss the latest studies for intrinsically stretchable (*is*-) electronic/optoelectronic materials, including the formation of conductive nanocomposite and polymeric blending with various additives. The review also provides examples of *is*-LEDs, focusing on their luminous performance and stretchability. We conclude this review with a brief outlook on future technologies.

## 1. Introduction

Over the past few decades, the primary focus in the development of information displays has been on elevating image quality to afford users realistic and immersive visual experiences. Extensive research efforts have been devoted to innovating display hardware, and critical parameters for device performance such as resolution, dynamic range, frame rate, and color reproducibility have undergone significant advancements. The evolution of display hardware, coupled with the advent of transformative technologies like the Internet of Things (IoT), has given rise to the concept of the “interactive display”. Wirelessly linked to other electronic devices, an interactive display can serve as a versatile interface between the user and the machine [1,2]. For example, displays for extended reality (XR) visualize information collected from various sources like surrounding appliances or wearable sensors, enabling users to actively engage with their environment [3]. 

A key objective of interactive displays is to foster an increased intimacy between users, promoting improved mobility, user convenience, and real-time interactivity. In this regard, deformable displays featuring soft and flexible form factors have emerged as alternatives to conventional rigid displays, offering mechanical adaptability with various objects, particularly with soft human tissues [4,5,6,7]. For electroluminescent (EL) devices in deformable displays, thin device thickness is essential to minimize the strain applied on active layers during bending deformation [8]. The development of high-performance light-emitting diodes (LEDs), such as inorganic micro-LEDs (*μ*-LEDs) [9,10], organic LEDs (OLEDs) [11,12], and quantum dot LEDs (QLEDs) [13,14], have enabled the development of flexible displays based on their thin film structures [15,16]. Such ultrathin LEDs are either transferred or directly deposited onto flexible substrates [17,18], exhibiting high operational stability when subjected to bending deformation. Based on the active matrix (AM) driving of LED arrays [19,20], flexible displays have been successfully manufactured and already brought to the commercial market. Following the successful launch of the Samsung “Galaxy Z Fold” in 2019, numerous global display manufacturers have unveiled their plans to pioneer the next generation of flexible displays, encompassing multi-foldable, slidable, and rollable displays [6]. 

Beyond the advancements in flexible displays, current research initiatives are now concentrating on displays endowed with stretchability [21,22]. Stretchable displays, capable of maintaining their luminous performance under the reversible elongation and relaxation, are expected to create unprecedented user experiences, especially in various human-friendly wearable electronics [23,24]. For instance, integrated with stretchable electronics like wearable sensors, actuators, and electrical circuits, these stretchable displays can visualize the collected bio-information to users even in scenarios involving crumpling, elongation, or being attached to human skin with dynamic fluctuation [25]. 

Imparting stretchability to LEDs, however, poses more challenging issues compared to the fabrication of flexible LEDs. The strategy commonly employed in the fabrication of flexible displays—reducing the thickness of the device—may not be particularly effective in the fabrication of stretchable LEDs [26]. When subjected to substantial amount of strain, severe cracks would be generated in active layers of LEDs, inducing device failure [27]. To address this issue, the development of stretchable LEDs has relied on additive strategies, which are categorized into two approaches: “structural approaches” and “material-based approaches,” based on their distinctive mechanisms to tolerate applied strain [28,29].

**Figure 1:**
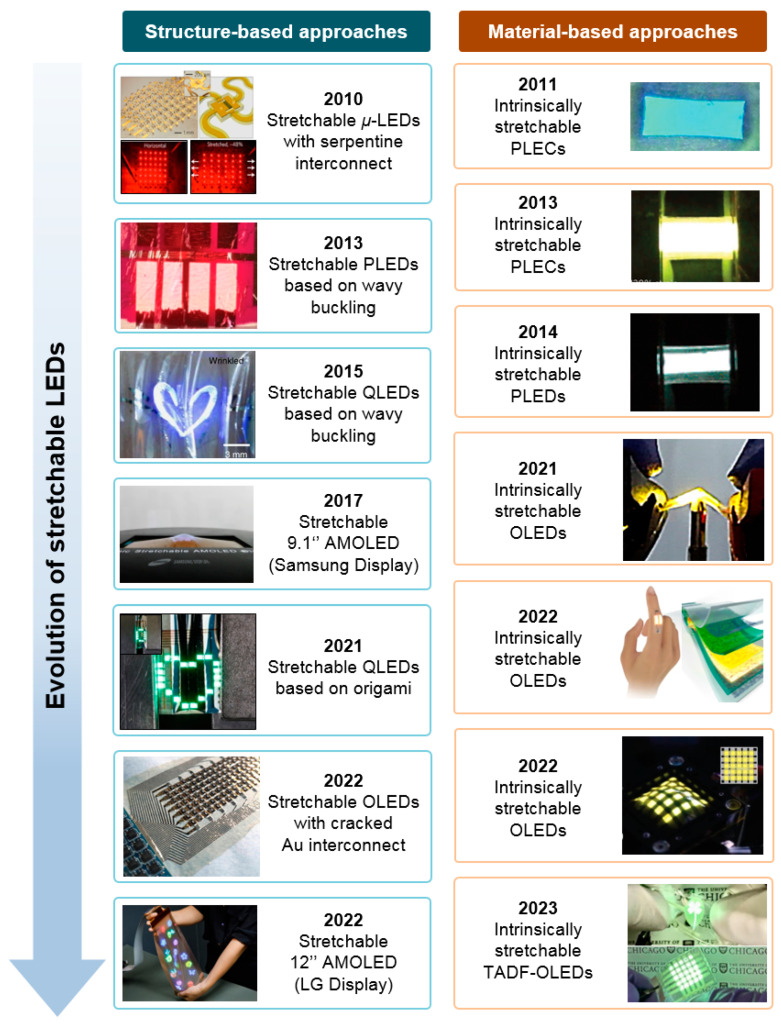
A brief chronology of the evolution of stretchable LEDs based on structural and material-based approaches, respectively. Reproduced with permission from Ref. [30], Copyright 2010, Springer Nature. Reproduced with permission from Ref. [31], Copyright 2013, Springer Nature. Reproduced with permission from Ref. [32], Copyright 2015, Springer Nature. Reproduced with permission from Ref. [33], Copyright 2017, Society for Information Display. Reproduced with permission from Ref. [34], Copyright 2021, Springer Nature. Reproduced with permission from Ref. [35], Copyright 2017, AAAS. Reproduced with permission from Ref. [36], Copyright 2023, Society for Information Display. Reproduced with permission from Ref. [38], Copyright 2011, Wiley-VCH. Reproduced with permission from Ref. [39], Copyright 2013, Springer Nature. Reproduced with permission from Ref. [40], Copyright 2014, American Chemical Society. Reproduced with permission from Ref. [41], Copyright 2021, AAAS. Reproduced with permission from Ref. [42], Copyright 2022, Springer Nature. Reproduced with permission from Ref. [43], Copyright 2022, Wiley-VCH. Reproduced with permission from Ref. [44], Copyright 2023, Springer Nature.

Figure 1 illustrates the evolution of stretchable LEDs over the past decade, following these two approaches. The structural approach is based on the mechanically engineered device structures to effectively distribute the strain [30,31,32,33,34,35,36]. With such geometries, researchers have succeeded in creating stretchable EL devices even when utilizing LEDs that are not naturally stretchable [37]. Based on this approach, global display manufacturers, such as Samsung Display or LG Display, have showcased the prototype of stretchable AM-OLED panels [33,36]. The material-based approach involves creating intrinsically stretchable (*is*-) LEDs exclusively using soft and stretchable materials [38,39,40,41,42,43,44]. Conventional non-stretchable electronic materials should be replaced to *is*-conductors and *is*-semiconductors [45,46,47,48]. Consequently, a sophisticated material-processing technology has been developed to guarantee the high mechanical, electrical, and optoelectronic performance of such materials [49,50].

In this review, we discuss recent advancements in both structural and material-based approaches for creating stretchable LEDs. Initially, we present representative device designs aimed at efficient strain distribution, incorporating island-bridge structures, wavy buckling, and kirigami/origami-based designs. For material-based approaches, we examine the latest research on *is*-electronic/optoelectronic materials, including the development of conductive nanocomposites, integration with various additives, and molecular modifications in polymers. The review also highlights the examples of *is*-organic LEDs, emphasizing their current luminous performance and stretchability. To conclude, we briefly discuss future technological prospects.

## 2. Structural Approaches for the Fabrication of Stretchable LEDs

Materials used in traditional LEDs include transparent conducting oxides (TCOs) and metal thin films for their electrodes as well as various organic/inorganic semiconductors for their light-emissive and charge transport layers [51,52]. Typically, these materials are vacuum-deposited onto rigid substrates, such as glasses or wafers, featuring a highly uniform, crystalline morphology that ensures high carrier mobility. Employing these materials with outstanding electronic properties, LEDs with high luminous performance and operational stability can be achieved. Conversely, the inherent rigidity and brittleness of these materials, often characterized by a high Young’s modulus (>10 GPa) and low crack-onset strain (<1%), make them susceptible to mechanical deformation, especially for stretching [53].

**Figure 2:**
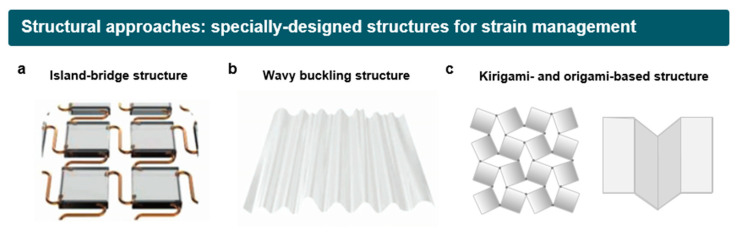
Structural approaches based on specially designed structures. (**a**) Rigid island-bridge structures with serpentine interconnects. (**b**) Prestrain-induced buckling structure. (**c**) Kirigami- and origami-based structure.

In this regard, to fabricate stretchable EL devices with non-stretchable components, unique device architectures have been widely explored to effectively dissipate applied strain (Figure 2). Examples of these architectures include the island-bridge structure with serpentine interconnects, wavy buckling, and kirigami-/origami-inspired structures. These designs have the capability to localize applied strain away from the active layers, enabling stretchable EL devices to sustain their luminous performance during repeated elongation and relaxation [54]. The major advantage of this approach lies in the leveraging of the advantages of established LED technologies, guaranteeing the high luminous performance and operational stability of stretchable EL devices. In this section, we discuss representative design strategies for stretchable LEDs with a specific focus on three distinctive device designs and their respective mechanisms for managing strain.

### 2.1. Stretchable LED Arrays Based on the Island-Bridge Structure

Stretchable LED arrays with the island-bridge structures generally consist of non-stretchable LED parts of “islands” and stretchable interconnect parts of “bridges” [55,56]. LED islands are arranged in a checkerboard pattern on an elastomeric substrate, and the bridges connecting each island are composed of stretchable interconnects capable of maintaining conductivity under the applied strain. This architecture has been devised to isolate tensile strain from the rigid LEDs during stretching while accommodating significant strain [57]. The majority of the strain is localized in the interface between the rigid LEDs and soft elastomers [58].

*Μ*-LEDs have emerged as the predominant option for stretchable EL devices employing island-bridge structures, which is attributed to their exceptional efficiency, brightness, and long-term stability. Facilitated by significant advancements in fabrication technology, the chip sizes of *μ*-LEDs have continuously reduced to the microscale (dimensions of 100 μm or even smaller), exhibiting their potential for high-resolution displays. The typical device structure of *μ*-LED is based on the p-n junctions of epitaxial semiconductors, leveraging the recombination of injected charge carriers at the junction interface. All internal components of a *μ*-LED consist of inorganic materials, which are impervious to humidity, leading to the remarkable longevity of LEDs (device lifetime > 100,000 h).

The first stretchable *μ*-LED array, based on the rigid-island bridge structure with a geometrically designed interconnect, was reported by Rogers et al. [30]. Kim et al. reported the stretchable *μ*-LED array composed of 36 pixels of AlGaInP *μ*-LED, which exhibited deep red emission. Each LED pixel was connected with the serpentine-shaped interconnect, which was composed of Au thin films sandwiched between two polyimide (PI) layers. The interconnect was patterned by photolithographical methods. The interconnect served as both electrical interconnection and structural bridges between each LED pixel. The serpentine-shaped interconnect effectively mitigates the strain applied to the pixels during the stretching of the array. In this regard, the devices demonstrated the ability to withstand 48% uniaxial stretching without device failure.

**Figure 3:**
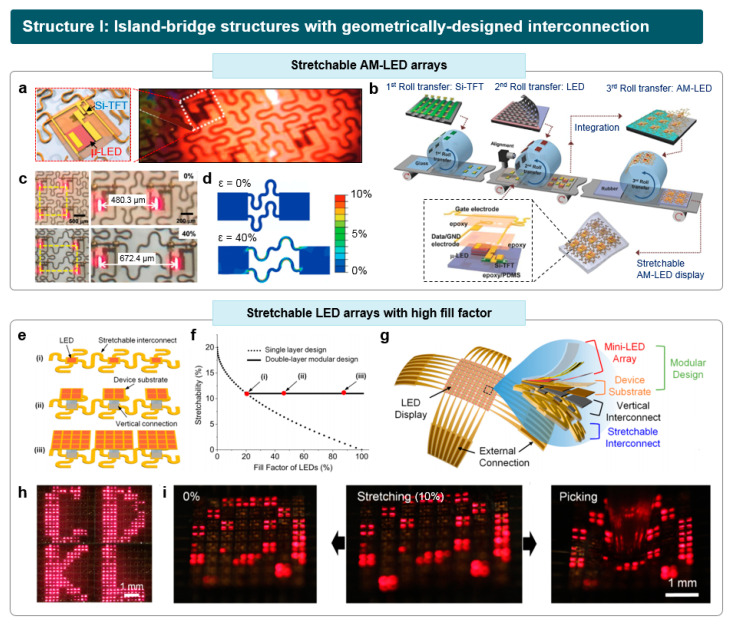
Stretchable LED arrays based on island-bridge structures with geometrically designed interconnect. (**a**) Stretchable AM-LED arrays composed of Si-TFT and *μ*-LEDs. (**b**) Roll transfer printing process for the fabrication of stretchable AM-LED arrays. (**c**) Optical images of stretchable AM-LED arrays under 0 and 40% of uniaxial strain. (**d**) Strain distribution on the stretchable AM-LED display, simulated by FEM. Reproduced with permission from Ref. [59], Copyright 2017, Wiley-VCH. (**e**) Stretchable inorganic LED arrays using the double-layer modular design. (**f**) Theoretical fill factor in a single-layer design (dotted line) and a double-layer modular design (solid line) with respect to the applied strain. (**g**) Schematic illustration of the stretchable LED arrays with an exploded view. (**h**) Optical images of the stretchable LED array, displaying alphabets. (**i**) Mechanical deformation of the stretchable LED array (left, initial state), such as 10% stretching (middle) and picking by a tip (right). Reproduced with permission from Ref. [61], Copyright 2022, American Chemical Society.

Subsequently, numerous studies have been reported to fabricate stretchable LED arrays using similar approaches, striving for progress in materials, structural design, and device performance. Choi et al. reported the fabrication of stretchable *μ*-LED arrays with AM operation, comprising 64 pixels of a single crystalline Si thin film transistors (TFT) and an AlGaInP *μ*-LED (Figure 3a) [59]. Each pixel was connected with a serpentine-shaped interconnect. Si-TFT and *μ*-LEDs were initially fabricated on rigid source substrates, ensuring wafer-level electronic and optoelectronic performance. Through the overlay-aligned roll transfer printing process, the high-performance Si-TFT and *μ*-LEDs were integrated on the rubber substrate, preserving their original performance (Figure 3b). The electron mobility of the released Si-TFT was over 700 cm^2^ V^−1^ s^−1^, and the on–off ratio is as high as 10^6^. Also, the transfer-printed *μ*-LEDs exhibited stable performance under a low voltage range (~4 V) comparable to their bulk properties.

Although the Si-TFTs and *μ*-LEDs exhibited high, reliable electrical and optoelectronic properties, the poor mechanical properties of inorganic materials limit the mechanical range of the resulting EL devices. To deal with the issue, the island-bridge structure with serpentine-shaped interconnects was applied, resulting in the fabrication of the stretchable EL devices while maintaining high device performance (Figure 3c). According to the mechanical analysis on the strain applied to the devices (Figure 3d), most of the applied strain was focused on the serpentine bridges, especially for the inner part of the serpentine interconnect and the interfaces between the bridge and island. In contrast, the island region experiences a minimal amount of strain (<0.1%), ensuring the stable operation of Si-TFT and *μ*-LEDs. In comparison to passive matrix (PM) operation, which sequentially addresses each pixel in a row–column scheme at a high frequency, the fabricated stretchable AM-LED arrays exhibited low power consumption, showcasing high potential for next-generation stretchable displays.

One of the major drawbacks of the island-bridge structure with serpentine interconnects lies in the limitation in fill factor [60,61]. The fill factor is characterized as the proportion of the light-emitting active area covered by LED cells to the total area of the system. In island-bridge structures, a notable trade-off exists between the overall stretchability of the system and the fill factor. This is because only the interconnect regions undergo stretching, while the pixel area remains unaltered. In this regard, conventional methodologies had to sacrifice the fill factor to achieve the sufficient stretchability of the system, leading to low pixel density and luminance of the arrays. Such increased dark regions between the LEDs pose a challenge for the practical application of stretchable displays based on this approach, especially for high-resolution displays.

To address the trade-off between the fill factor and stretchability, research has been conducted on modulating the size of individual LED cells. Kim et al. reported the stretchable mini-LED arrays with a high fill factor, which was enabled by the double-layer modular design coupled with larger size of LED cells [62]. As shown in Figure 3e, case (ii) and case (iii) have a double-layer modular design with an increased size of single LED cells. While case (i) with a conventional single-layer modular design exhibits a limited fill factor of 20%, case (iii) achieved high fill factors of 90% at the same stretching condition (Figure 3f). Between the modular design of mini-LED arrays and stretchable interconnect layers, a vertical interconnect was made via the soldering process in order to electrically connect the mini-LED to a stretchable interconnect (Figure 3g). The contact areas and via holes were formed in the device, using a dry-etching process. The stretchable mini-LED PM arrays demonstrated significantly enhanced areal coverage compared to the conventional single-layer design (Figure 3h). These arrays exhibited robust device stability when subjected to stretching or picking by a tip (Figure 3i).

Another challenge of the island-bridge structures accompanied with serpentine interconnects lies in their relatively low mechanical stability [63,64]. The mechanical mismatch between the rigid and soft region may lead to the mechanical failure at their interface where the majority of the applied strain is concentrated. Also, the out-of-plane buckled structure of brittle interconnects may not be suitable for practical displays, given that most display panels are encapsulated with flat glasses. Thus, the research trend for stretchable interconnects has moved toward the use of *is*-conductors to achieve mechanical robustness. The *is*-conductors, such as liquid metals [65,66], conductive polymers [67,68], and nanocomposites [69,70], are extensively investigated as printable conductive inks for stretchable interconnects. These materials demonstrate the ability to preserve their conductivity under applied strain without the need for additional design modifications.

### 2.2. Stretchable LEDs Based on Prestrain-Induced Wavy Buckling

Inducing a wavy buckling structure on ultrathin LED is an another prevalent structural approach for the fabrication of stretchable EL devices. In this approach, thin film LEDs, such as OLEDs [8,31], QLEDs [32,71], or perovskite LEDs [72], are initially deposited onto a flexible film substrate, such as PI, polyethylene terephthalate (PET), or polyethylene naphthalate (PEN). The total thickness of the flexible LEDs, including the substrate, active layers, and top encapsulation layer, is generally in the range of several tens of micrometers, exhibiting high mechanical flexibility. Then, the flexible LEDs are transferred onto a prestrained elastomer substrate. Upon releasing the prestrain, the flexible LEDs undergo compressive strain from the elastomer substrate, creating periodic wavy buckling. The bending radii of buckling is significantly affected by the thickness of the ultrathin LEDs and mechanical properties of elastomers. As the material is stretched, these wrinkles progressively flatten until the elastomer substrate expands to approximately the prestrain level. In contrast to island-bridge structures, which demonstrate a notable trade-off between fill factors and stretchability, stretchable LEDs utilizing the buckling approach do not face limitations in fill factors, as stretching occur evenly in both the active emission area and the interconnection parts.

The key part in this approach lies in the strain management for a generated buckling structure. Given that a long bending radius can lead to severe image distortion, it is generally recommended to strive for a small bending radius in buckling. Consequently, maintaining the integrity of active materials becomes imperative due to the reduced bending radii associated with each generated buckling, often measuring only several tens of micrometers. Ensuring that the strain experienced by active materials during bending deformations remains below the crack-onset strain of each material is critical. As a concise approximation, the applied strain (*S*) on a thin-film device with thickness (*h*) and bending radius (*R*) can be expressed as *S* = *h*/2*R* [73]. In this regard, reducing the total thickness of flexible LEDs is the most critical parameter to reduce the strain applied to brittle materials, thus guaranteeing the high mechanical stability.

Also, the device structure to minimize the strain applied on the active layers has been widely explored. When a structure undergoes bending deformation, compressive strains are applied to the inside, while tensile strains affect the outside of the device. Meanwhile, a specific plane situated in the middle of the structure, referred to as the neutral mechanical plane, undergoes nearly zero net strains. Therefore, placing active layers composed of high modulus materials on the neutral mechanical plane can mitigate device failures caused by material damage, such as fractures. By optimizing the thickness of top/bottom passivation layers (or substrates), the location of neutral mechanical planes can be adjusted [74,75].

**Figure 4:**
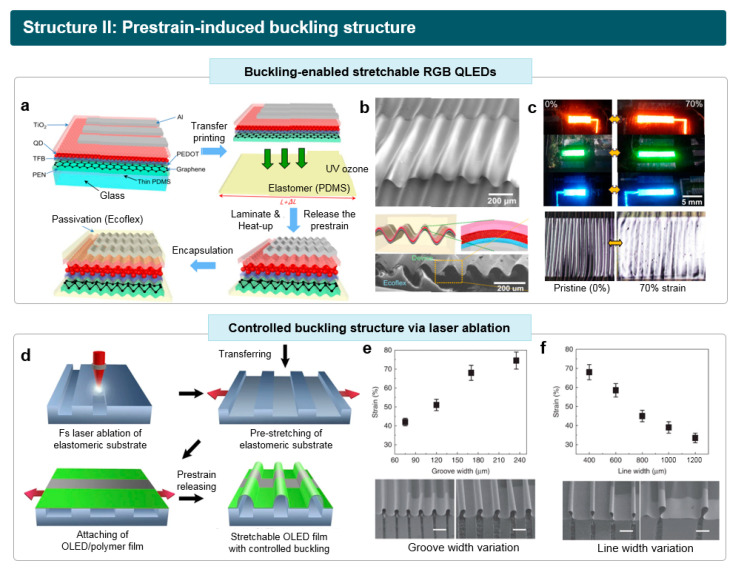
Stretchable LEDs based on prestrain-induced wavy buckling structure. (**a**) Schematic illustration of the fabrication process to create stretchable QLEDs with wavy buckling. (**b**) Scanning electron microscope (SEM) image of the generated buckling (upper panel), and schematic illustration (lower panel) of the generated wavy buckling. (**c**) 70% uniaxial stretching of RGB QLEDs. Reproduced with permission from Ref. [76], Copyright 2022, American Chemical Society. (**d**) Schematic illustration of the fabrication process to achieve controlled wavy buckling via laser-assisted pre-programming of the substrate. (**e**) The effect of grove width on maximum stretchability of the buckling structure. SEM images in the bottom exhibit buckling structures with different groove widths. (**f**) The effect of line width on the maximum stretchability of the buckling structure. SEM images in the bottom exhibit buckling structures with different line widths. Reproduced with permission from Ref. [77], Copyright 2022, Springer Nature.

Based on the buckling formation approach, the stretchable full-color (RGB) QLEDs was reported by Kim et al. [76]. Figure 4a demonstrates the fabrication process of buckling-enabled stretchable QLEDs. Ultrathin QLEDs were initially fabricated on a 1.3 μm thick PEN substrate. The ultrathin QLED has a device structure of graphene (anode)/poly(3,4-ethylenedioxythiophene)poly(4-styrenesulfonate) (PEDOT:PSS, a hole injection layer (HIL))/poly[(9,9-dioctylfluorenyl-2,7-diyl)-co-(4,4′-(N-(4-s-butylphenyl))diphenylamine)] (TFB, a hole transport layer (HTL))/CdSe QDs (an emissive layer (EML))/TiO_2_ (an electron transport layer (ETL))/Al (cathode). Note that the ITO electrode was replaced to four-layered graphene to achieve higher strain tolerance and mechanical stability in this research. The ultrathin QLED was then transfer-printed onto a prestrained PDMS substrate. After releasing the prestrain, periodic wavy buckling was created on the device, and the crumpled device was encapsulated by a stretchable passivation layer. The bending curvature of the generated periodic buckling was 35 μm (Figure 4b). By controlling the thickness of the passivation layer, the active layer of the QLED, comprising various non-stretchable materials, was positioned on the neutral mechanical plane. This strategy minimized material damage, enabling the device to withstand bending deformation. The buckling-enabled stretchable QLEDs with RGB light emission can be stretched up to 70% without significant loss of luminous performance (Figure 4c).

Under identical prestrain levels, the maximum elongation of buckling-enabled stretchable LEDs can be controlled by tuning the 1D gratings on the elastomeric substrate. Yin et al. reported the stretchable OLEDs with buckling structures, wherein the structure can be pre-programmed using the laser ablation technique on an elastomeric substrate (Figure 4d) [77]. Using a femtosecond laser with the emission profile of 800 nm peak wavelength, 100 fs pulse width and 1000 Hz repetition rate, the microstructure was fabricated on the elastomer substrate of 3 M VHB tape. The width and depth of the grooves was regulated by adjusting the laser intensity. Then, the flexible OLED with the total thickness of around 10 μm was transferred on the 120% prestained elastomers, generating the buckling structure after releasing the prestrain. The maximum elongation of the stretchable OLED increases as the groove width widens with a constant line width of 400 μm (Figure 4e). Conversely, the maximum strain decreases with an increase in line width, maintaining a fixed groove width of 170 μm (Figure 4f). Upon comparing variations in groove and line widths before and after stretching, it revealed that the stretching predominantly occurred in the grating grooves where the buckles rise. Based on this tunable buckling structure, a highly efficient stretchable OLED with 70 cd A^−1^ of current efficiency was fabricated, exhibiting 70% of stretchability.

### 2.3. Stretchable LEDs Based on Kirigami- and Origami-Inspired Structures

Recently, design strategies inspired by paper artwork, named kirigami and origami, have been extensively applied to a wide range of electronics, encompassing antennas [78], image sensors [79,80], battery [81,82], and bioelectronics [83,84]. A kirigami-based structure involves cutting the edges or central parts of devices [85,86], while an origami-based structure involves the controlled folding of devices into desired patterns [87,88,89]. When applied to stretchable LEDs, kirgami and origami-inspired structures can provide a reliable route for transforming the 2D plane-wise LED structures into various 3D forms under the applied external forces [90,91,92]. A sophisticated design strategy involving the pre-programmed cutting and folding patterns facilitates the effective dissipation of external strain during deformation. This includes the isolation of the majority of strain away from the rigid active layers or utilizing neutral mechanical planes.

The major challenge in this technique is in the development of a facile and reliable fabrication process to pre-program the LEDs to generate cutting and folding patterns without degrading the device performance of LEDs. The conventional dry-/wet-etching process, accompanying harsh plasma and/or various organic solvents, may damage the active organic/inorganic layers of LEDs. In this regard, laser ablation methods have been extensively investigated for pre-programming the devices based on their ability to precisely ablaze the desired regions and facilitate large-area processing with time efficiency. By controlling the laser spec, such as an emission wavelength, irradiance, and frequency, the etching profiles such as the width and depths of the etched region can be customized [93].

**Figure 5:**
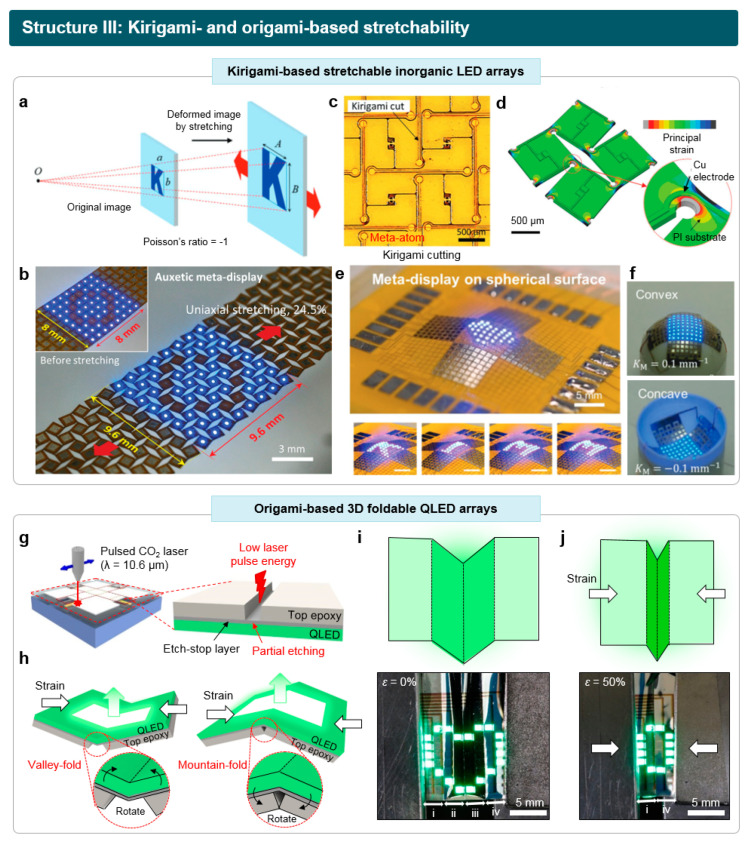
Stretchable LEDs based on kirigami- and origami-based structures. (**a**) Uniaxial stretching of an auxetic meta-display without image distortion. (**b**) Schematic image of the stretchable auxetic meta-display, based on kirigami. (**c**) Optical image of the stretchable auxetic meta-display. Cutting patterns are pre-programmed via laser ablation. (**d**) Strain distribution inside the auxetic meta-display during stretching, calculated using FEM. (**e**) Operation of the stretchable auxetic meta-display attached on spherical substrate. (**f**) Meta-display attached to convex (upper panel) and concave (lower panel) surfaces. Reproduced with permission from Ref. [94], Copyright 2022, Wiley-VCH. (**g**) Pre-programming of 3D foldable QLEDs via selective laser-etching process. (**h**) Folding deformation of the pre-programmed device. By applying a compressive force, the partially etched folding line can be deformed into either a valley-fold or a mountain-fold. (**i**,**j**) Schematic (upper images) and photographs (lower images) of the origami-based stretchable QLED array. When the device undergoes compression, the initial representation of a heart shape (**i**) transforms into ‘0’ (**j**), as the inner two sub-panels become obscured from view. Reproduced with permission from Ref. [34], Copyright 2022, Wiley-VCH.

Jang et al. reported the stretchable *μ*-LED array, achieving low image distortion by utilizing a kirigami-based circuit board for its substrate [94]. As most stretchable displays are located on an elastomeric substrate with a Poisson’s ratio near 0.5, the internal shape of these stretchable devices may undergo significant image distortion, such as the compression in the direction perpendicular to the applied strain. To deal with the issue, they designed a kirigami-based auxetic substrate to achieve the Poisson’s ratio near −1. With this metamaterial, the *μ*-LED arrays can stretch while preserving the geometric similarity of the original shape even under the uniaxial strain (Figure 5a,b). The kirigami cutting patterns were pre-programmed on devices to achieve a negative Poisson’s ratio [95], which can be tuned by designing the unit cell structures (Figure 5c). The cutting patterns were formed via a laser ablation technique on circuit boards, using an ultraviolet (UV) laser with a wavelength of 266 nm. The kirigami-based substrates experienced an expansion in the perpendicular direction, featuring the rotation of unit cells under the applied uniaxial strain. According to the finite element analysis methods (FEM) to calculate the strain distribution, the majority of strain was found to be concentrated on the hinge region, connecting each unit structure (Figure 5d). By integrating the kirigami-based substrate with *μ*-LED arrays, a meta-display showcasing a negligible image distortion during stretching was successfully demonstrated and applied to convex and concave spherical structures (Figure 5e,f).

Also, an origami-based stretchable LED was fabricated by pre-programming of the device with folding and cutting patterns using laser ablation. Kim et al. reported the fabrication of a 3D foldable QLED based on an origami-inspired structure, exhibiting the reversible transformation between various 2D to 3D shapes under the applied strain [32]. A selective laser etching technique was developed using unique device structures with etch-stop layers. Based on the high etching thresholds of a silver layer against the CO_2_ laser with an emission wavelength of 10.6 μm, only the upper epoxy layer with a lower laser threshold was selectively etched (Figure 5g). Introducing thinner segments on the ultrathin LEDs enables the devices to be sharply folded along the laser-etched lines in two directions—mountain-folds and valley-folds—under applied forces (Figure 5h). Based on this technique, a curtain-shaped, 3D foldable QLED array with 64 pixels was fabricated. The device demonstrated facile stretching and compression, allowing for dynamic transformations between 2D planar shapes and various 3D non-planar structures (Figure 5i,j).

Kirigami- and origami-inspired structures hold great promise for stretchable displays, and discussions are ongoing regarding their potential application in commercial display products. However, meeting the stringent stability criteria, such as undergoing numerous cyclic tests for repetitive deformations in commercial products (>10^5^ cycles), poses a formidable challenge. Repetitive exposure to strain can lead to irreversible degradation in materials concentrated in areas where strain is localized, potentially causing a gradual deterioration in active pixels or interconnections located in those regions. Therefore, research in mechanical engineering, such as exploring novel kirigami and origami structures to effectively distribute strain, must be complemented by further studies in material engineering that enhance the strain tolerance of component materials to achieve high mechanical stability.

## 3. Material-Based Approaches for the Fabrication of Stretchable LEDs

As an alternative to the structural approach, material-based approaches have been extensively explored to fabricate stretchable LEDs. In this approach, *is*-LEDs, capable of maintaining their luminous performance under stretching, are fabricated on elastomeric substrates. To replace conventional rigid materials for LED components, including electrodes, EMLs, and charge transport layers, *is*-electronic materials should be newly developed.

**Figure 6:**
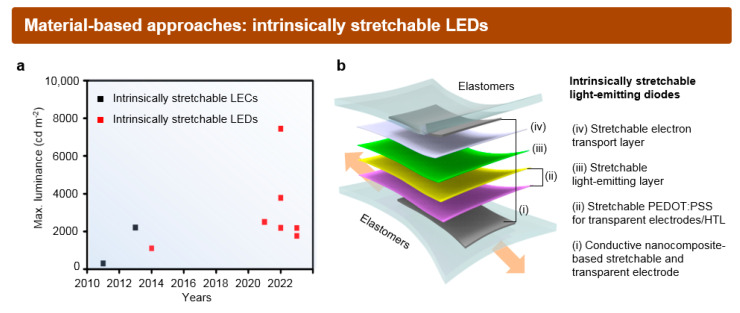
Material-based approaches for the fabrication of *is*-LEDs. (**a**) The improvement in the maximum luminance of reported *is*-LEC and *is*-LED over the past decade. (**b**) Schematic illustration of the representative device structure of *is*-LEDs.

In contrast to the island-bridge structure, where the fill factor undergoes a notable reduction during the stretching process, *is*-LEDs maintain their fill factors without experiencing a decrease. The expansion of the active emission area in *is*-LEDs occurs concurrently with the extension of the interconnect region. This stands in stark contrast to the structural approach, which frequently encounters mechanical failures at the strain-localized interface between the rigid LED region and stretchable interconnect region; *is*-LEDs ensure superior mechanical durability by incorporating soft materials for all components. The first *is*-light-emitting electrochemical cell (LEC) was reported by Pei et al. in 2011 [38]. Note that the stretchable LEC, featuring a device structure that includes an EML sandwiched between two stretchable electrodes, is classified within the broader category of material-based approaches for stretchable LEDs. This classification is based on similarities between two devices, such as emitting light under direct current (DC) driving conditions. Stretchable alternating current-driven EL devices (ACELs) are thus excluded from this category [96,97]. Driven by the technological advancements in material-processing and device fabrication technology, the performance of *is*-LEDs has been significantly increased (Figure 6a). The detailed information for the previously reported *is*-LEDs, such as their device structures, luminous performance, and maximum stretchability, are provided in Table 1 [38,39,40,41,42,43,44,98,99,100].

The representative device structure of such *is*-LEDs is provided in Figure 6b. For transparent and stretchable electrodes, conductive nanocomposites based on the percolation network of 1D nanomaterials (i) or modified conducting PEDOT:PSS for enhanced stretchability (ii-1) have been widely used. These electrodes are either embedded or deposited on the elastomer substrate. For stretchable HTLs (ii-2), modified semiconducting PEDOT:PSS has been generally employed based on its high hole mobility. In the case of *is*-EML (iii), the primary method for achieving stretchability has typically involved integrating light-emitting polymers with organic additives. For stretchable ETL (iv), n-type semiconducting polymers, such as poly(9,9-bis(3′-(N,N-dimethyl)-N-ethylammoinium-propyl-2,7-fluorene)-alt-2,7-(9,9-dioctylfluorene)) (PFN) or polyethylenimine ethoxylated (PEIE), have been employed. As there are only a few studies reporting on the research progress on (iv), our main focus in this section will be on recent studies concerning the development of components (i), (ii), and (iii) of *is*-LEDs. While discussing (iii), we will collectively address the current development level of state-of-the-art *is*-LEDs, encompassing the major aspects like luminance performance and maximum stretchability.

### 3.1. Conductive Nanocomposite for Stretchable and Transparent Electrodes

Conductive nanocomposites, comprising conductive nanofillers embedded in an elastomer matrix, have emerged as one of the most extensively studied materials for stretchable and transparent conductors [101,102]. The electrical characteristics of conductive nanocomposites primarily depend on the material properties of the conductive nanofillers. It is crucial for these nanofillers not only to exhibit high conductivity individually but also to form a mesh-like percolation network. The percolation network created by conductive nanofillers within an elastomer matrix can facilitate the movement of electrons both through the nanofillers themselves as well as the contact junctions between nanofillers. The higher the number of unbroken contact junctions maintained during elongation, the greater the conductivity achieved when strain is applied. Also, due to the mesh structure of conductive fillers, light can penetrate through the voids, allowing high optical transmittance.

The percolation theory has been developed as a powerful analysis method for predicting the formation of a conductive network embedded in an elastomer matrix [103,104,105]. One of the key parameters for the calculation is the composition of the nanocomposite. When the contents of nanofiller in the composite surpass the percolation threshold, the minimum contents required for the formation of a percolation network, conductive pathways are established based on the generated contact junctions. Thus, higher contents of nanofillers beyond the percolation thresholds result in a significant increase in the conductivity. However, loading an excessive amount of nanofillers can result in a deterioration of the overall mechanical performance of elastomers, impacting factors such as strain durability and mechanical toughness. The interference of an excessive quantity of nanofillers with the crosslinking between elastomer chains can lead to insufficient stretchability. Additionally, the optical transmittance of the nanocomposite significantly decreases with higher concentrations of nanofillers, as the voids become filled with nanofillers. Therefore, the weight ratio between the nanofillers and elastomer contents should be carefully tuned to achieve the optimized mechanical/electrical/optical properties of nanocomposites.

The dimension and size of nanoparticles are also important parameters to determine the properties of nanocomposite. Among various conductive nanomaterials, 1D nanomaterials such as metallic nanowires have been the most suitable option for conductive nanofillers, as they have the smallest percolation thresholds. Compared to nanomaterials with 0D nanoparticles or 2D nanoflakes, these 1D materials can achieve high levels of conductivity with a relatively small amount of fillers, avoiding issues caused by high loading of the filler [106,107,108]. Specifically, percolation networks created by nanowires with high aspect ratios exhibit enhanced conductivity when compared to those with low aspect ratios, as the formation of network structures using longer nanowires requires fewer contact junctions. The contact resistance between each nanowire occupies the significant portion of total resistance of nanocomposite. In this regard, since nanowires with high aspect ratios require less fractional volume to attain equal conductivities compared to nanowires with low aspect ratios, their nanocomposites typically yield additional advantages in terms of trade-off characteristics. These may include improved stretchability and/or higher transmittance.

**Figure 7:**
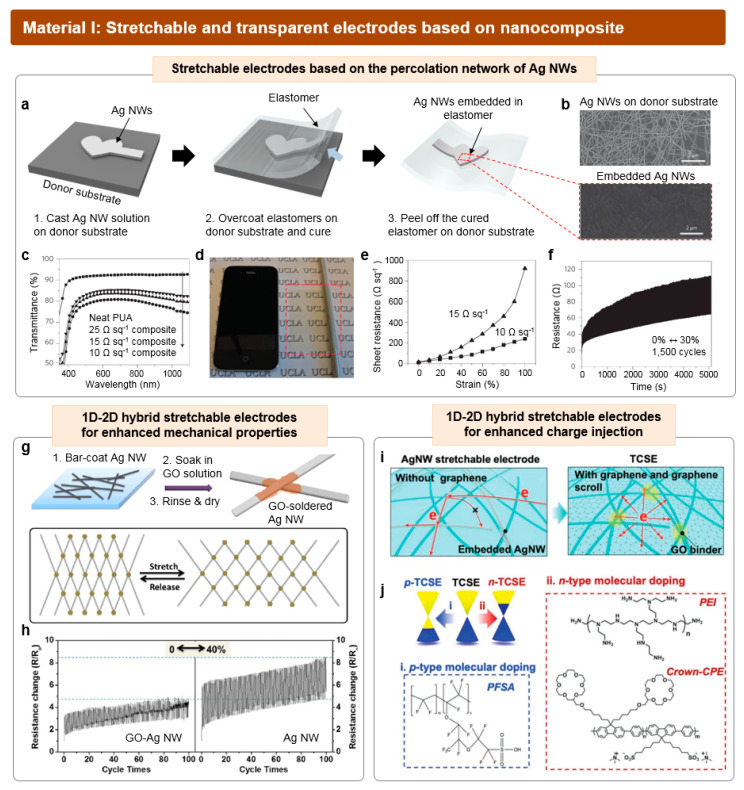
Stretchable conductive composite based on silver nanowire network for stretchable and transparent electrodes. (**a**) Schematic images of the fabrication process to create free-standing Ag NW-embedded stretchable electrodes. (**b**) SEM images of the Ag NWs directly coated on the donor substrate (upper panel) and Ag NWs embedded in elastomer matrix (lower panel). (**c**) Optical transmittance of the Ag NW-embedded stretchable electrodes. (**d**) An image exhibiting the transparency of the Ag NW-embedded stretchable electrode (a red-dashed square). (**e**) Sheet resistance–strain curve of two Ag NW-embedded stretchable electrodes with different initial sheet resistance. (**f**) Variations in resistance during a repeated 30% stretching test for up to 1500 cycles. Reproduced with permission from Ref. [39], Copyright 2013, Springer Nature. (**g**) Strengthening of the contact junctions in the Ag NW network via graphene oxide soldering. The reinforced network exhibits higher mechanical stability under stretching (lower image). (**h**) Variations in resistance of graphene oxide-soldered electrodes (left) and non-soldered electrodes (right), during a repeated 40% stretching test. Reproduced with permission from Ref. [40], Copyright 2014, American Chemical Society. (**i**) Schematic images illustrating the charge injection properties at the surface of Ag NW-embedded stretchable electrodes without (left) or with graphene scrolls (right). (**j**) p-type and n-type dopant material for doping the Ag NW-embedded stretchable electrodes with graphene scrolls. Reproduced with permission from Ref. [43], Copyright 2022, Wiley-VCH.

The direct coating of a nanocomposite onto the elastomeric substrate, such as the spray coating or spin coating of nanocomposite solution, may lead to the electrodes with a bumpy surface, resulting in uneven light-emission or short-circuiting of LEDs. To deal with the issue, an unconventional fabrication process was reported to achieve the uniform surface roughness (Figure 7a). After the deposition of silver nanowires (Ag NWs) on the donor substrate, the UV-curable elastomer was poured on the substrate and cured. Then, the free-stranding, stretchable and transparent electrodes are peeled off from the donor substrate. As shown in Figure 7b, the majority of Ag NW volumes are embedded in the elastomeric matrix, exhibiting the smooth surface of electrodes [39]. Due to the presence of void volume between nanowires, the stretchable electrodes exhibited high optical transmittance (~80%, Figure 7c,d). Furthermore, these stretchable electrodes were able to sustain their conductivity even when subjected to 100% uniaxial stretching or subjected to cyclic stretching for 1500 cycles at 30% strain (Figure 7e,f).

Hybrids of heterogeneous nanomaterials have been also employed as conductive nanofillers. In particular, combinations involving 1D nanowires and 2D nanoflakes have been extensively reported, providing additional benefits to nanocomposites. For instance, Liang et al. reported the development of stretchable and transparent electrodes with enhanced stretchability achieved through the soldering of contact junctions in the percolation network with graphene oxides (Figure 7g) [40]. The contact junctions between each Ag NW were reinforced with the attached GO nanoflakes, exhibiting higher mechanical stability under the repeated elongation and strain releasing process. Consequently, the network soldered with graphene oxides exhibited a smaller change in resistance during cyclic stretching under 40% strain in comparison to the control group (Figure 7h).

Additionally, the incorporation of 2D conductors can significantly improve the charge injection into adjacent semiconducting organic layers. Zhou et al. reported stretchable electrodes based on hybrids of Ag NWs and graphene scrolls embedded in an elastomer matrix [43]. Conventional stretchable electrodes utilizing pristine Ag NWs face limitations due to a restricted contact area confined to the 1D Ag NW/organic layer interface. In contrast, the graphene scroll enhances the dispersion of charge carriers, resulting in higher carrier injection efficiency. (Figure 7i). Moreover, the work function of the hybrid electrodes has been modified by introducing appropriate dopants (Figure 7j). For p-type dopants, perfluorosulfonic acid (PFSA) was used, while polyethyleneimine (PEI) or anionic crown conjugated polyelectrolyte (crown-CPE) were used for n-type dopants. The introduction of these dopants has facilitated the proper alignment of energy levels with the adjacent semiconducting layers. This enhancement in charge injection properties has led to the superior luminous performance of the resulting *is*-OLEDs.

Despite considerable research efforts toward developing stretchable and transparent nanocomposites based on silver nanowires, the stability and reliability of metallic nanowires in ambient conditions remain challenging due to silver’s susceptibility to oxidation. Coating the surface of Ag NW with gold presents a viable solution for achieving higher stability [109,110,111]. Another critical issue in the nanocomposite is the restricted stretchability of electrodes coupled with a significant rise in resistance during stretching. Several studies have aimed to alleviate the rise in resistance during stretching, employing approaches like engineering the surface ligands of nanofillers to finely adjust the interfacial interaction between nanofillers and elastomers [112,113].

### 3.2. Additive-Blended PEDOT:PSS for Stretchable Electrodes and Hole Transport Layers

Conductive conjugated polymers, such as PEDOT:PSS, have been widely used for stretchable and transparent electrodes as well as *is*-HTLs for the fabrication of *is*-LEDs. The ratio between positively charged conjugated PEDOT contents and negatively charged insulating PSS contents largely determines the electrical properties of PEDOT:PSS, ranging from conductors to p-type semiconductors. As pristine conductive PEDOT:PSS generally exhibits poor conductivity (~1 S/cm) as well as low stretchability (<20% of crack-on-set strain), various post-treatment or additive-blending methods have been reported to enhance both conductivity and stretchability [114,115,116]. In most cases, a solution of modified PEDOT:PSS is directly coated on elastomeric substrate to form a polymeric thin film. For stretchable electrodes, the deposited PEDOT:PSS film should be patterned to define the light-emitting area [117].

**Figure 8:**
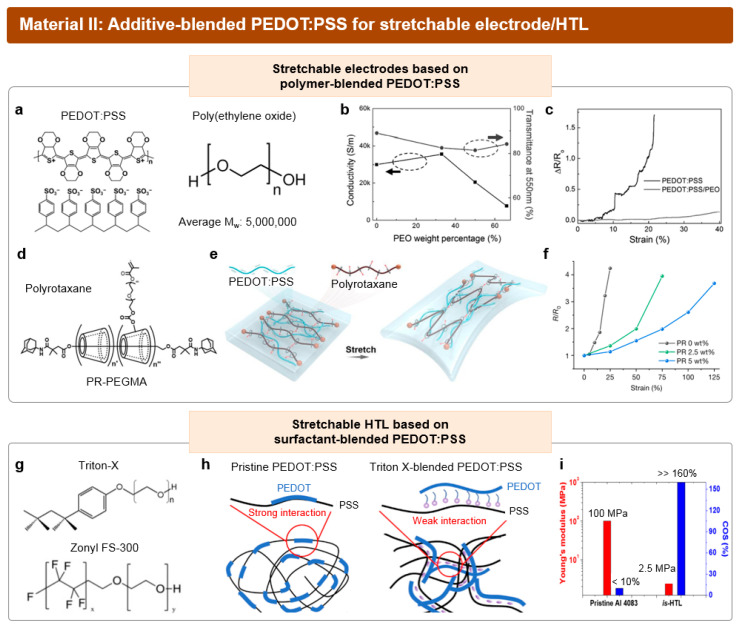
Additive-blended PEDOT:PSS for stretchable and transparent electrodes and stretchable HTL. (**a**) Molecular structures of PEDOT:PSS (left) and PEO (right). (**b**) Variations in conductivity and transmittance with varying weight fractions of PEO. (**c**) Changes in the resistance of pristine PEDOT:PSS and PEO-blended PEDOT:PSS under stretching. R_0_ and R represent the resistance before and after stretching, respectively. Reproduced with permission from Ref. [118], Copyright 2017, Wiley-VCH. (**d**) Molecular structure of the polyrotaxane. (**e**) Schematic illustration of stretching of the PR-blended PEDOT:PSS electrodes. (**f**) Changes in the resistance of pristine PEDOT:PSS and PEO-blended PEDOT:PSS under stretching. Reproduced with permission from Ref. [42], Copyright 2022, Springer Nature. (**g**) Molecular structures of Triton X and Zonyl FS-300. (**h**) Schematic illustration of phase separation of the pristine PEDOT:PSS (left) and Triton X–blended PEDOT:PSS (right). Triton X weakens the electrostatic interaction between the PEDOT region and PSS region, imparting stretchability to HTL. (**i**) Changes in Young’s modulus and crack-onset strain of PEDOT:PSS with respect to the amount of Triton-X. Reproduced with permission from Ref. [41], Copyright 2021, AAAS.

A heterogeneous polymer composite of PEDOT:PSS and other polymers has been the most widely explored method to fabricate stretchable PEDOT:PSS electrodes. Reported by Bade et al., PEDOT:PSS was blended with high-molecular-weight poly ethylene oxide (PEO, M_w_ = 5,000,000) in aqueous solution (Figure 8a) [116]. Thin films with varying PEO contents were achieved via spin coating of the blended solution on PDMS substrate, resulting in 200 nm thick electrodes. The conductivity of the electrodes exhibited an initial increase upon the addition of PEO, which was followed by a subsequent decrease at higher PEO concentrations (Figure 8b). According to the Raman spectroscopy, the rise in conductivity at lower PEO concentrations (<30 wt%) may be attributed to a conformational change in the PEDOT:PSS from a benzoid structure (a coil-shaped form) to a quinoid structure (a linear form). To simultaneously achieve both outstanding electrical and mechanical properties, the composition of the blend was carefully adjusted. The PEO-blended PEDOT:PSS film achieved a peak conductivity of 35,600 S m^−1^ with a PEO content of 33 wt% while maintaining high optical transmittance of 82%. The PEO-blended PEDOT:PSS film (33 wt% of PEO) was capable of maintaining its conductivity until 40% of strain without a significant rise in resistance (Figure 8c).

Recently, Zhang et al. reported a highly conductive, stretchable, and transparent electrode based on the combination of PEDOT:PSS and supramolecules (Figure 8d) [42,119]. Polyrotaxane (PR) is a supramolecular material which has a structure of threading linear molecules through numerous cyclic compounds. Polyethylene glycol (PEG) and cyclodextrin serve as representative materials for linear molecules and cyclic compounds, respectively. While PEG is known for enhancing the conductivity of PEDOT:PSS through the aggregation of the PEDOT phase, its high crystallinity may lead to severe phase separation, which compromises the stretchability. The cyclic compounds in PR play a crucial role in reducing the crystallinity of PEG, ensuring high stretchability of the composite as well as high conductivity (Figure 8e). Also, the side chains in cyclic compounds enable the photo-patterning of stretchable electrodes, which is facilitated by UV light and photomasks. With 5 wt% of PR content, the PR-blended PEDOT:PSS electrode demonstrated a high transmittance of 92% at 550 nm, which is capable of stretching up to 125% with only a fourfold increase in resistance (Figure 8f).

On the other hand, a surfactant-blended semiconducting PEDOT:PSS (Clevios AI 4083, Heraeus Epurio Clevios™, Dayton, OH, USA) has found extensive use in *is*-ETL [41]. Triton X and Zonyl FS-300, both serving as surfactants with hydrophobic heads and hydrophilic tails in their molecular structures, are commonly employed surfactants for this purpose (Figure 8g). These materials act as plasticizers, weakening the electrostatic interaction between PEDOT and PSS through a charge-screening effect. They also induce a phase separation of PEDOT:PSS, resulting in the formation of a nanofibrous PEDOT structure (Figure 8h). The phase separation of semiconducting PEDOT:PSS blended with Triton X resulted in superior stretchability when compared to pristine PEDOT:PSS. With 5 wt% of Triton X, the blend exhibited a significant decrease in Young’s modulus from 100 to 2.5 MPa as well as a dramatic increase in crack-onset strain from 10 to 160% (Figure 8i).

### 3.3. Intrinsically Stretchable Light-Emitting Layers Based on Polymers

While other EL emitters, such as inorganic semiconductors, quantum dots, organic small molecules, and perovskites, have rigid and brittle film morphology, light-emitting polymers exhibit mechanical ductility with relatively low crystallinity. In this regard, light-emitting polymers have been predominantly used for EL emitters of *is*-LEDs. A representative example of a light-emitting polymer for stretchable EML is SuperYellow (SY), which is a commercially available, yellow-emitting conjugated polymer based on poly(1,4-phenylenevinylene) (PPV) derivatives. Despite the dynamic slipping or migrating of SY polymer chains contributing to its mechanical ductility, the pristine SY exhibits insufficient stretchability, which is characterized by a limited crack-onset strain of 30 to 40% or even less. Thus, enhancing crack-onset strain and stretchability has been a focus in research, which is often achieved by blending additives with light-emitting polymers. Paralleled with the methodologies employed in the fabrication of stretchable conductive polymers, such as additive-blended PEDOT:PSS, the major challenge of this approach lies in the careful optimization of the composition. As many additives are electrically insulating, a trade-off between the electrical properties and mechanical properties is generally aroused.

**Figure 9:**
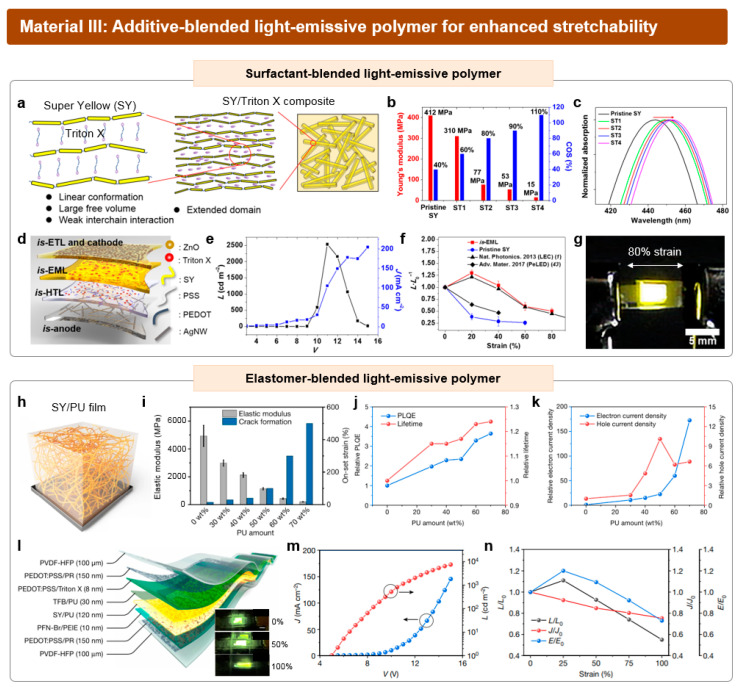
Additive-blended light-emissive polymers for intrinsically stretchable light-emitting layer. (**a**) Schematic illustration of the internal morphology of SY/Triton X composite. (**b**) Changes in Young’s modulus and crack-onset strain of SY with respect to the amount of Triton X. (**c**) Changes in absorption peak wavelength after the blending of Triton X. (**d**) Device structure of the fabricated *is*-OLED, utilizing SY/Triton X as an *is*-EML. (**e**) *J–V–L* characteristics of *is*-OLED. (**f**) Changes in luminance of the reported device using *is*-EML (red line) and pristine SY (blue line) and two previously reported references (two black lines) with respect to strain. L_0_ and L represent the luminance before and after stretching, respectively. (**g**) Photograph of the 80% stretched *is*-OLED. Reproduced with permission from Ref. [41], Copyright 2021, AAAS. (**h**) Schematic illustration of the SY/PU composite for *is*-EML, featuring a nanofiber of SY inside the PU matrix. (**i**) Changes in Young’s modulus and crack-onset strain of *is*-EML with respect to the amount of PU. (**j**) Relative PL efficiency and PL lifetime of SY/PU films with increased PU amount. (**k**) Relative electron and hole current densities of SY/PU films with increased amount of PU at 8 V. (**l**) Device structure of the fabricated *is*-OLED, utilizing SY/PU as an *is*-EML. The inset shows the stretching of the *is*-OLED from 0 to 100% strain. (**m**) *J–V–L* characteristics of the *is*-OLED. (**n**) Changes in luminance (black line), current density (red line) and current efficiency (blue line) of the reported *is*-OLED, during stretching. J_0_ and J represent the current density before and after stretching, respectively. E_0_ and E represent the current efficiency before and after stretching, respectively. Reproduced with permission from Ref. [42], Copyright 2022, Springer Nature.

Kim et al. reported the fabrication of *is*-organic EML by incorporating a non-ionic surfactant named Triton X to SY [41]. As shown in Figure 9a, the addition of Triton X induced the weakening of the interchain interaction between SY polymer chains, thereby increasing the free volume inside the conjugated polymers. In this regard, the mechanical properties of the blended composite are significantly affected by the weight fraction of Triton X. As the weight ratio of Triton X increased from 0 to 1:2 of SY:Triton X, the Young’s modulus exhibited a gradual decrease from 412 to 15 MPa, while the crack-onset strain concurrently increased from 30 to 110% (Figure 9b). Interestingly, the absorption wavelength of the composite was red-shifted as more Triton X was added (Figure 9c). This phenomenon is attributed to the extension of the conjugation length of the SY chains. While the pristine SY exhibit a coiled, spherical domain based on the strong interaction between the rigid conjugated segments, the SY/Triton X composite has a linear conformation with an extended conjugation length of SY chains. The composition of the *is*-EML was optimized at a ratio of 2:1 for SY:Triton X, as an excessive weight fraction of Triton X led to a significant decrease in carrier mobility.

The optimized *is*-EML was readily applied to the fabrication of *is*-OLED (Figure 9d). For its stretchable and transparent anode and *is*-HTL, the aforementioned Ag NW-based conductive nanocomposite and Triton X-blended PEDOT:PSS were used, respectively. For *is*-ETL, the composite solution of ZnO nanoparticles and PEIE was spin coated on the *is*-EML, exhibiting no crack formation under 100% strain. The stretchable cathode is deposited on the *is*-ETL via spray-coating Ag NW dispersion. *is*-ETL was coated again on the Ag NW cathode to fully embed the Ag NW network inside the *is*-ETL, resulting in the enhanced electron injection capacity. The maximum luminance of the *is*-OLED was 2500 cd m^−2^ at 11 V, while the turn-on voltage of the device was 8 V (Figure 9e). When the device was subjected to uniaxial strain, it maintained 50% of its original luminance when stretched up to 80% strain (Figure 9f,g). Replacing the *is*-EML with pristine SY brought a notable decrease in luminous performance, which was likely attributed to the formation of severe cracks or voids in the pristine SY.

An alternative approach for imparting sufficient stretchability in *is*-EML involves blending light-emitting polymers with elastomers. Recently, an *is*-EML based on the polymeric composite of SY and polyurethane (PU) was reported by Zhang et al. [40]. The composite has an interpenetrating network of SY, featuring a uniformly distributed SY chain inside the elastomeric PU matrix (Figure 9h). While severe phase separation occurred in the composite of SY and elastomer with non-polar segments (SEBS), the uniform morphology of the SY/PU composite was likely attributed to the polar group interactions between SY and PU. As the weight proportion of PU increased from 0 to 70 wt%, the Young’s modulus of the *is*-EML decreased from 5 GPa to 205 MPa, and the crack-onset strain significantly increased from 30 to 500% (Figure 9i).

The photoluminescence (PL) performance of the SY/PU composite shows an increasing trend as the weight fraction of PU increases to 70 wt% (Figure 9j). This is ascribed to the suppression of trap-assisted non-radiative decay of SY through blending, which was strongly supported by the enhanced electron and hole transport abilities even with an increase in the insulating PU content. In electron-only device data, measuring trap-limited electron transport, the current level significantly increased with the addition of more PU in the composite (Figure 9k). Nonetheless, it is crucial to avoid an excessively high PU ratio to prevent a shortage of luminescent material. The optimal EL performance was observed at an approximately 30% PU ratio.

The optimized SY/PU composite film was readily applied to the fabrication of *is*-OLEDs (Figure 9l). All components of the *is*-OLEDs have exhibited decent stretchability while maintaining their original electrical performance. PR-blended PEDOT:PSS was deposited on the elastomer substrate of poly(vinylidene fluoride-co-hexafluoropropylene) (PVDF-HFP) via spin coating, which was utilized for both the stretchable cathode and anode. The stretchable electrodes exhibited outstanding stretchability, conductivity, and transparency at the same time. Triton X-blended PEDOT:PSS and the mixture of TFB and PU were employed as *is*-HIL and *is*-HTL, respectively. For *is*-ETL, the polymeric composite of PFN-Br and PEIE was used. The fabricated *is*-OLED achieved a maximum luminance of 7450 cd m⁻² at 15 V, marking the highest record among all previously reported *is*-OLEDs (Figure 9m). The device retained 50% of its original luminance under 100% stretching (Figure 9n).

Introducing insulating additives to light-emitting polymers is an easily applicable approach for achieving stretchability, but it may also entail a significant trade-off between optoelectronic properties and mechanical properties. To address this issue, alternative methodologies have been explored recently, which exclude the use of additives. Reported by Liu et al., light-emitting polymers with high quantum efficiency and intrinsic stretchability were newly synthesized through a sophisticated engineering of the polymer chain [44]. Their design strategy involves incorporating flexible, linear units into a polymer backbone [120,121,122], which can significantly enhance mechanical stretchability without compromising the underlying electroluminescent processes. As a result, the reported *is*-EML was able to be stretched up to 125% of strain even without the incorporation of additives.

## 4. Conclusions

In this review, we provide an overview of the recent progress on both structural and material-based approaches for fabricating stretchable LEDs. For structural approaches, we presented representative device designs aimed at efficient strain distribution, covering island-bridge structures, wavy buckling, and kirigami-/origami-based structures. For material-based approaches, we discussed the latest studies for the fabrication of high-performance *is*-LEDs, mainly focusing on the development of *is*-electronic/optoelectronic materials, including conductive nanocomposites based on 1D nanomaterials and/or additive-blended conducting/semiconducting polymers.

Due to the advantages of fully leveraging established LED technologies, stretchable displays based on structural designs are approaching commercialization. Nevertheless, persistent challenges, particularly in addressing the operational durability of stretchable displays, remain. In the case of buckling structures, the consistent exposure of compressive/tensile strain on active layers may lead to the degradation of device performance, resulting in a significant decrease in the long-term stability of LEDs. Island-bridge structures or kirigami-based designs, which utilize the strain dissipation to deformable interconnections/hinge regions, also face challenges in long-term durability. As the majority of strain is localized to interconnections/hinges, there may be a gradual increase in interconnection resistance or short circuits in soldered regions, adversely affecting the long-term stability of devices. To deal with the stability issues, conductive materials capable of withstanding substantial amounts of strain are essential for interconnections or hinges. Conductive materials deposited on traditional flexible substrates are inadequate for accommodating such large strains. Instead, stretchable conductors with elastomeric properties should be employed for interconnections or hinges to tolerate deformation.

On the other hand, *is*-LED technology is still in its early stages of development with ample room for improvement. The luminous performance of the reported *is*-LEDs lags behind that of conventional rigid or flexible LEDs. For example, the record value of maximum luminance among all previously reported *is*-OLEDs is only 7450 cd m^−2^, even when operating at a high voltage of 15 V [42]. Moreover, the operational lifetime of *is*-LEDs typically spans only a few hours or even less, which is significantly below the standards required for commercialization. The insufficient performance and stability of *is*-LEDs may have complex origins, including confined carrier injection from stretchable electrodes and inefficient carrier transport at the bulk and/or interface of *is*-electronic materials [123]. Also, the limited encapsulation ability of elastomers is also a critical issue attributed to the low operation stability of *is*-LEDs [124,125].

Furthermore, the development of a facile fabrication process is essential, considering that most stretchable functional layers rely on solution-based materials [126]. While the cost advantage of solution processing over vacuum deposition is evident, the challenge lies in the necessity for a reliable assembly method of materials, such as a printing-based deposition method. Attaining a high-resolution patterning of stretchable electrodes or *is*-EML down to sub-micrometer pixel sizes is also crucial for their effective application in full-color *is*-AM-LED arrays. We anticipate that significant advancements in *is*-LEDs with high performance and longevity will drive significant advancements in deformable display technology. This includes the development of next-generation free-form displays leveraging high-resolution AM arrays of full-color *is*-LEDs.

## Figures and Tables

**Table 1:** Previously reported *is*-OLECs and *is*-OLEDs. Device structures, composition of *is*-EMLs, luminous performance, and device stretchability are provided for each reference.

Device Type	Year	Device Structures		*is*-EML Composite		Max. Luminance (cd m^−2^)	Turn-on V (V)	Max. Stretchability (%)	Ref.
				Emitters	Additives				
*is*-OLEC	2011	Anode	SWNT-PtBA	PF-B (65 wt%)	PEO-DMA (32 wt%) LiTf (3 wt%)	300 (@12 V)	4.8	45	[38]
		Active layers	*is*-EML						
		Cathode	SWNT-PtBA						
	2013	Anode	Ag NW-PUA/ PEDOT:PSS	SuperYellow (80 wt%)	ETPTA (8 wt%) PEO (8 wt%) LiTf (4 wt%)	2200 (@21 V)	6.8	120	[39]
		Active layers	*is*-EML						
		Cathode	Ag NW-PUA						
	2020	Anode	Ag NW-PUA/ PEDOT:PSS	SuperYellow (46.5 wt%)	IC polymer (46.5 wt%) ETT-15 (4.7 wt%) LiTf (2.3 wt%)	N/A	15	30	[98]
		Active layers	*is*-EML						
		Cathode	Ag NW-PUA						
*is*-OLED	2014	Anode	GO-Ag NW-PUA	White-emitting polymer (91 wt%)	OXD-7 (9 wt%)	1100 (@21 V)	7.0	130	[40]
		Active layers	PEDOT:PSS/ *is*-EML/PEI						
		Cathode	GO-Ag NW-PUA						
	2021	Anode	Ag NW-PDMS	SuperYellow (67 wt%)	Triton-X (33 wt%)	2500 (@11 V)	8.3	80	[41]
		Active layers	PEDOT:PSS-Triton X/*is*-EML/ZnO-d-PEIE						
		Cathode	Ag NW-PDMS						
	2022	Anode	Graphene-Ag NW-SEBS	SuperYellow (80 wt%)	Triton-X (20 wt%)	2185 (@15 V)	5.3	73	[42]
		Active layers	PEDOT:PSS-Triton X/*is*-EML/Crown-CPE						
		Cathode	Graphene-Ag NW-SEBS						
	2022	Anode	Au	L-SY-PPV (50 wt%)	PAN (50 wt%)	3780 (@13 V)	6.5	30	[99]
		Active layers	PEDOT:PSS-Triton X/*is*-EML/PMMA/Zn-PEIE-pBphen-TR						
		Cathode	Ag/Ag NW-PDMS						
	2022	Anode	PEDOT:PSS-PR	SuperYellow (50 wt%)	PU (50 wt%)	7450 (@15 V)	5.0	100	[42]
		Active layers	PEDOT:PSS-Triton X/TFB-PU/*is*-EML/PFN-Br-PEIE						
		Cathode	PEDOT:PSS-PR						
	2023	Anode	Graphene-Ag NW-SEBS	SuperYellow (59 vol%)	PEO (35 vol%) KCF_3_SO_3_ (6 vol%)	1754 (@11 V)	3.7	30	[100]
		Active layers	PEDOT:PSS-Triton X/*is*-EML						
		Cathode	Graphene-Ag NW-SEBS						
	2023	Anode	Ag NW-TPU-PDMS	PDKCD (100 wt%)	None	2175 (@10 V)	4.7	60	[44]
		Active layers	PEDOT:PSS-PFI/*is*-EML/PEIE-PFN-Br						
		Cathode	Ag NW-TPU-PDMS						
